# Clinicopathologic Characteristics of Gallbladder Adenomyomas and the Contribution of Macroscopic Sampling in Adenomyoma Diagnosis

**DOI:** 10.5146/tjpath.2019.01471

**Published:** 2020-01-15

**Authors:** Selma Şengiz Erhan, Sevinç Hallaç Keser, Mehmet Özer

**Affiliations:** Department of Pathology, University of Health Sciences, Okmeydani Education and Research Hospital, Istanbul, Turkey; Department of Pathology, University of Health Sciences, Kartal Dr. Lutfi Kirdar Education and Research Hospital, Istanbul, Turkey; Bursa Yuksek Ihtisas Education and Research Hospital, Bursa, Turkey

**Keywords:** Gallbladder, Adenomyoma, Macroscopic sampling

## Abstract

***Objective:*** Adenomyoma, a reactive and hamartomatous lesion of the gallbladder, is included in the differential diagnosis of several benign and malignant lesions. Macroscopic sampling is very important in the determination of these lesions. The agreed macroscopy protocol in recent years has been prepared by the Hepatopancreatobiliary Pathology Working Group. We aimed to evaluate the clinicopathologic properties of adenomyoma cases in the gallbladder and the contribution of new macroscopy techniques to the diagnosis of adenomyoma in the pre-protocol and post-protocol parts of a one-year period.

***Material and Method:*** Two institutes were included in the study. Adenomyoma cases diagnosed in the pre-protocol and post-protocol periods of one year duration were included in the study. Slides and demographic properties of the cases were reexamined.

***Results:*** While adenomyoma was present in 22 of 1879 gallbladder before the protocol, it was observed in 32 of 1781 gallbladders in the post-protocol period. 17 of the cases were male and 37 were female. The mean age of the cases was 51.8. 52% of the lesions were located in the fundus. A gallstone was observed in 37 cases, and cholesterolosis in 14 cases. In the comparison of the two periods, the number of cases was lower in the post-protocol period but a 0.6% increase in the diagnosis of adenomyoma was found.

***Conclusion:*** Adenomyoma is one of the lesions of the gallbladder that should be recognized but can be easily overlooked macroscopically. When we conducted the sampling according to the last protocol, the increase in the diagnosis of adenomyoma showed that adequate and accurate sampling was very useful for the detection of adenomyoma in the gallbladder.

## INTRODUCTION

Adenomyoma is a hyperplastic lesion that is characterised by the proliferation of Rokitansky-Aschoff sinuses originating in the epithelial and accompanying muscular tissue (1). Lesions are subserous and the muscular layer is thickened. The incidence is 2-5%. Most cases are diagnosed between the ages of 50 and 60 (1,2). The symptoms are nonspecific; however, in very few cases, abdominal pain localized to the right upper quadrant may be the first symptom (3).

Although chronic irritation is reported to be an etiological factor, the pathogenesis of adenomyoma is controversial. A relationship with gallstone has frequently been reported (4). It can be easily overlooked macroscopically, and the diagnosis is generally possible microscopically (1,5).

Due to the lesions detected in the microscopic examination but that could not be detected in the macroscopic examination, the method and the required quantity of sampling from gallbladder material has been a subject of debate for many years (6,7). A macroscopic evaluation guide was prepared and the agreed macroscopy protocol was defined by the Turkish Federation of Pathology Societies, Hepatopancreatobiliary Pathology Working Group for the macroscopic examination and sampling method of the cholecystectomy materials (8). Before this protocol, gallbladders without a peculiarity were examined by one sample from each of the fundus, body and neck regions in our laboratory. Prior to this protocol, a sample of the fundus, body and neck of the gallbladder with no peculiarity was obtained at our clinic. After this protocol, longitudinal samples that contain the fundus, body and neck regions with the demonstration of the whole gallbladder wall were obtained. In this study, we aimed to evaluate the clinicopathological properties of adenomyoma cases which are difficult to recognize macroscopically and the contribution of a new macroscopic technique to the diagnostic process before and after the protocol.

## MATERIAL and METHOD

The cases with an adenomyoma diagnosis in the gallbladder with the samples taken and reported by different pathologists were included in the retrospective study conducted at two centres. The period covers one year before the protocol prepared by the Hepatopancreatobiliary Pathology Working Group (May 15, 2015-May 15, 2016) and one year following the protocol (May 16, 2016-May 16, 2017) in both of the clinics.

While in the pre-protocol period, gallbladders without a peculiarity were examined using one sample from each of the fundus, body and neck regions. In the post-protocol one year period, longitudinal samples obtained according to the protocol and that represented the whole area between the cystic duct surgical margin and fundus were exemplified in at least two cassettes. Some additional findings such as gender, age, adenomyoma localization, gallbladder dimensions, wall thickness in the lesional area, accompanying cholesterolosis, gallstone (single/multiple), metaplasia and dysplasia were also evaluated.

**Statistical Analysis: **In the comparisons between the categorical variables before and after the protocol, the parameters that met the chi-square test requirement were obtained with the chi-square test. Fisher exact test was performed for the parameters that did not meet the chi-square test condition. Numerical variables that represented a normal distribution were compared with the Student-t test. For parameters without a normal distribution, the Mann-Whitney U-test was used. The effect of gender, gallbladder size, and the presence of gallstones and cholesterolosis on the wall thickness was examined with linear regression analysis. The correlation between the wall thickness in the adenomyoma-containing area and gallbladder size was evaluated with the Spearman test. All the statistical analyses were performed with SPSS 17 (SPSS Inc., Chicago, USA) and p<0.05 was accepted as statistically significant.

## RESULTS

A total of 3660 gallbladder materials were present in the study period. A total of 54 adenomyoma cases were detected, with 22 cases (1.2%) of the 1879 gallbladders in the pre-protocol period and with 32 (1.8%) of the 1781 gallbladders in the post-protocol period. 17 of the cases were male (31.5%) and 37 (68.5%) were female. The age interval of the cases ranged from 31 to 73 years and the mean age was 51.81 ± 11.16 years.

**Figure 1:**
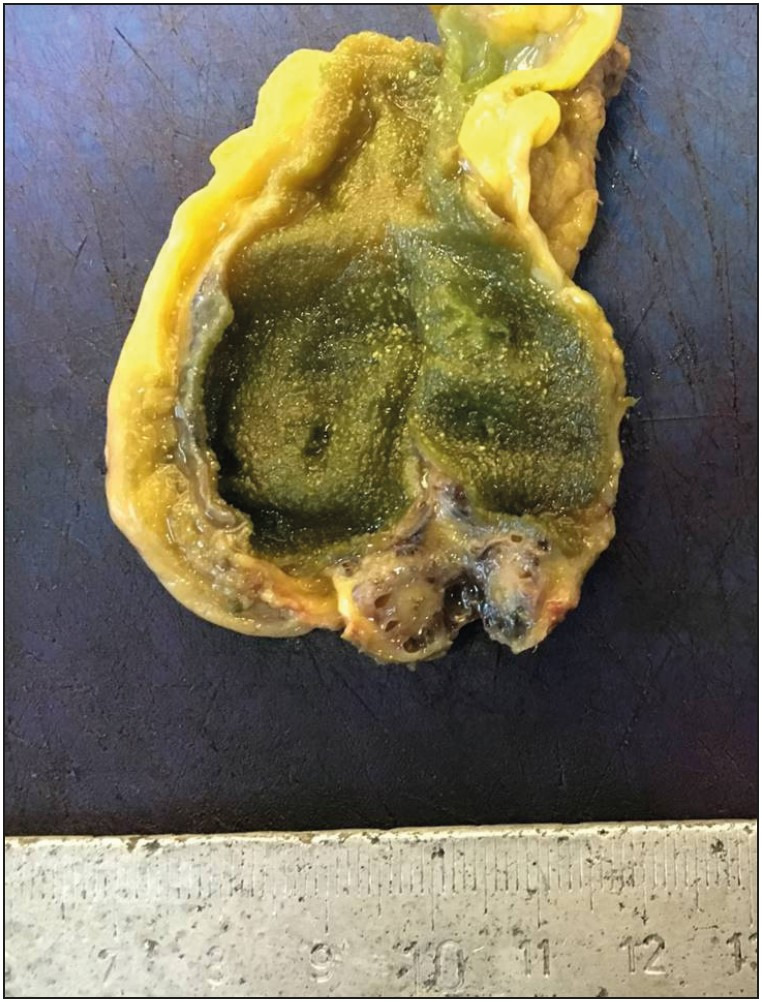
Fundal type adenomyoma; thickening of the gallbladder wall in the fundic region.

**Figure 2:**
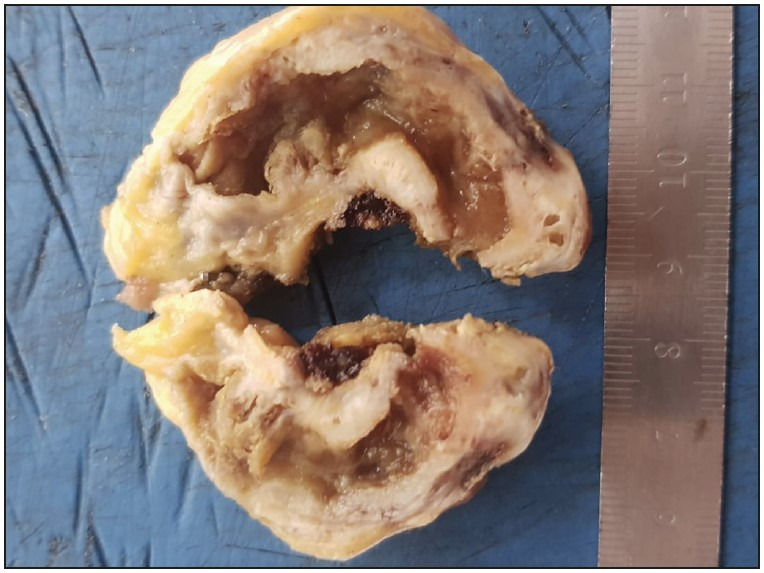
Segmental type adenomyoma; thickening of the gallbladder wall in the body and fundic region.

In the examination according to localization, 52 cases of adenomyoma (96.2%) were localized in the fundus and they were detected in one focus (Figure 1). In two cases (3.7%) diagnosed in the post-protocol period, adenomyoma with multifocal involvement (segmental type) including the fundus was present (Figure 2). The distance between the cystic duct surgical margin and the fundus ranged between 3.5 and 10.5 cm in the gallbladders. The wall thickness was over 2 mm in a total of 47 cases (87%), with 19 (86.3%) before the protocol and 28 (87.5%) following the protocol.

There was accompanying gallstones in 37 (68.5%) of the cases and accompanying cholesterolosis in 14 of the cases (25.9%). All of the adenomyoma cases accompanied by stone were fundus localized. The diameters of the gallstones ranged between 0.1 and 2.7 cm with an average of 0.82±0.65 cm. A single gallstone was found in 9 (24.3%) cases and multipe gallstones in 28 (75.7%) cases. A stone was not present in one of the segmental types of adenomyoma. In the second case in which the gallbladder was sent as opened, it was learnt from the surgeon that a stone was present. Yet, it was not possible to ascertain the information regarding the properties of the gallstone.

**Figure 3:**
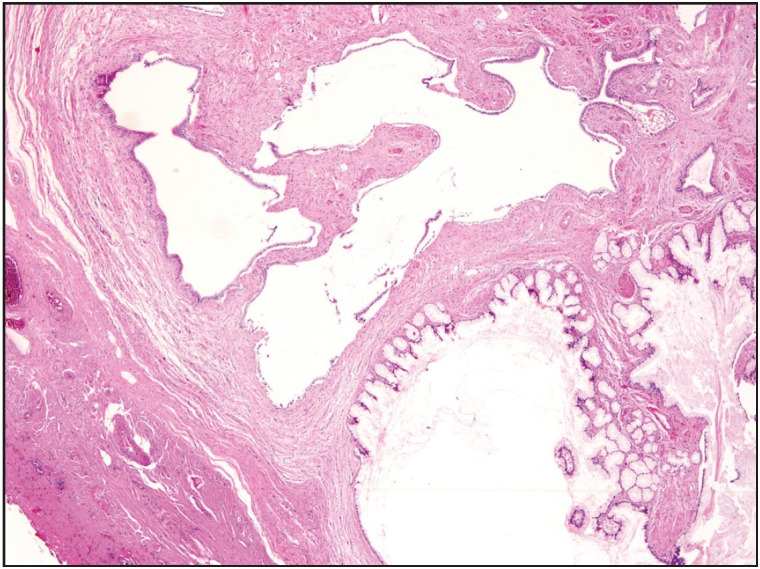
Fundal type adenomyoma. Cystically dilated glands and accompanying muscle bundles on the wall of the gallbladder. Pyloric metaplasia seen in the glandular component at the right lower corner (H&E; x40).

The adenomyoma was accompanied by a cholesterol polyp in 2 cases (3.7%), low-grade dysplasia in 2 cases (3.7%), pyloric metaplasia in 5 cases (9.3%) (Figure 3) and intestinal metaplasia in 3 cases (5.6%).

When the periods before and after the protocol were compared, there was no statistically significant difference between the adenomyoma diagnosis rates (p=0.11) that belong to the two periods. However, although the number of cases in the post-protocol period was less, an increase in the diagnosis of adenomyoma was observed at a rate of 0.6%. Segmental type adenomyoma diagnosis was present in two of the cases, both of which were in the post-protocol period.

The distribution in terms of age and gender of the cases was similar to each other in the pre-protocol and post-protocol period.

It was observed that the incidence of gallstone and cholesterolosis did not change depending on gender (p=0.51 and p=0.53, respectively). There was no significant effect of gender, gallbladder dimension, stone and cholesterolosis presence on the wall thickness in adenomyoma localized areas shown in Table 3 (p=0.43, p=0.18, p=0.54 and p=0.49, respectively). No significant difference was found between the dimensions of gallbladders that contain adenomyomas with a wall thickness of ≤ 2 mm or > 2 mm (p=0.69). No correlation was found between wall thickness and gallbladder dimensions (p=0.176, p=0.20). While the mean dimension of gallbladders that have adenomyoma with a wall thickness of ≤ 0.2 mm was 7.5 cm (7.0-9.0), this value was 7.5 cm (6.5-8.5) in gallbladders with adenomyomas with a wall thickness of > 2 mm (25-75th percentile values).

There was no significant difference in terms of accompanying stone and cholesterolosis rates (p>0.05) between the two periods but there was a statistically significant difference (p=0.03) in terms of intestinal metaplasia, pyloric metaplasia, cholesterol polyp and low grade dysplasia rates. These lesions were observed more often in the post-protocol period compared to the cases which were sampled before the protocol. The clinicopathologic findings and quantitative parameters of the cases are summarized in Table 1, Table 2 and Table 3.

**Table 1:** Comparison of pre-protocol and post-protocol parameters.

		**Pre-protocol**	**Post-protocol**	**p**
**Type**	Fundal	22 (100%)	30 (93.8%)	0.32^a^
	Segmental	0 (0%)	2 (6.2%)	
**Cholesterolosis**	No	17 (77.3%)	23 (71.9%)	0.66^b^
	Yes	5 (22.7%)	9 (28.1%)	
**Gallstone**	No	7 (31.8%)	10 (31.2%)	0.96^b^
	Yes	15 (68.2%)	22 (68.8%)	
**Gallstone count**	Single	3 (20%)	6 (27.3%)	0.71^a^
	Multiple	12 (80%)	16 (72.7%)	
**Accompanying findings**	No	22 (100%)	22 (68.8%)	0.03^a^
	Yes^#^	0 (0%)	10# (31.2%)	

^a^Fisher’s exact test ^b^Chi square test ^#^Five cases of pyloric metaplasia, three cases of intestinal metaplasia, two cases of cholesterol polyp and low grade dysplasia

**Table 2:** Quantitative parameters in the pre-protocol and post-protocol period.

	**Pre-protocol**	**Post-protocol**	**p**
**Gallbladder size**	7.5 ± 1.3	7.5 ± 1.4	0.99^a^
**Wall thickness**	0.5 (0.3-0.8)	0.4 (0.3-0.6)	0.60^b^
**Gallstone size**	0.5 (0.3-1.0)	0.5 (0.4-1.2)	0.43^b^

^a^Student t-test (Data have been expressed by mean value±standard deviation) ^b^Mann-Whitney U test (Data have been expressed as median (25-75th percentile))

**Table 3:** Results of multivariate linear regression analysis for the wall thickness in adenomyoma localized area.

**Factors**	**Coefficient**	**Standard error**	**95% Confidence interval**	**p**
**Gender**	0.072	0.091	0.111 to 0.255	0.43
**Gallbladder dimension**	0.046	0.034	0.114 to 0.022	0.18
**Stone presence**	0.057	0.093	0.130 to 0.243	0.54
**Cholesterolosis**	0.069	0.100	0.131 to 0.270	0.49

## DISCUSSION

The pathogenesis of gallbladder adenomyomas is still controversial. Degenerative disease caused by intraluminal pressure increase in the gallbladder due to an inability of bile discharge caused by motility failure is the most commonly accepted hypothesis (2,5,9). Other possible factors are thought to be gallstones, long lasting stimulation caused by chronic inflammation, estrogenic effect and the insufficiency of gallbladder budding in the embryonic period (3,9). As a result, epithelial intramural diverticules called Rokitansky-Aschoff sinuses and an accompanying hypertrophic muscular layer cause thickening of the gallbladder wall (1).

Adenomyoma is frequently seen between 50 and 60 years of age (5). There are differences in the literature about the incidence among men and women. While some authors have reported that it is three times more common in women, other authors reported similar rates in men and women (1,5). In our study, the age range is consistent with the literature. As adenomyomas have been reported to be two times more common in women than in men, we believe that an estrogenic effect could have a role in the etiopathogenesis (9).

In our study, the adenomyoma diagnosis rates we had detected in both pre-protocol and post-protocol periods were lower than those reported in the literature (1,2). We included in this study only the gallbladders with an adenomyoma diagnosis. On other gallbladders, adenomyoma may be absent in the diagnosis. This may lead to the assumption that this lesion was not be adequately sampled or not sufficiently recognized by pathologists working in the same or different departments. In addition, the differences and discrepancies in diagnosis and interpretation between the pathologists as regards adenomyomas in routine pathology reports may be another reason.

Normally, the long axis of the gallbladder is about 10 cm (10). In our cases, the range of the long axis was measured between 3.5 and 10.5 cm. In a vast majority of the healthy population, the thickness of the gallbladder wall is not more than 2 mm (5,10,11). 25% of the adenomyomas incidentally detected in the cholecystectomy materials of patients operated for other reasons such as stones and polyps usually have a wall thickness of more than 3 mm (5). In our study, cases with wall thickness of 4 mm or more were present, in parallel with the reports in the literature. We could not find any report or reference that investigated the relationship between the wall thickness and gallbladder dimension in the literature. There was no statistically significant relationship between these two parameters in our cases either.

Adenomyomas are divided into three groups based on their macroscopic properties (3,4): these groups are the fundal, segmental and diffuse types. The fundal type is frequently localized in the fundus and called a localized adenomyoma. In segmental adenomyomas, the gallbladder has an appearance similar that of an hour-glass, composed of fundal and neck compartments that have related lumens. The wall thickness in the fundal compartment is thicker than in the neck region due to the adenomyoma presence. In the diffuse type, the whole gallbladder wall is thickened (2). In some references in the literature, the fundal type (3,9) is stated to be the most frequently observed adenomyoma type. Yet, in other references, the segmental type (4,5) has been reported as the most frequent type. In our study, fundal type adenomyomas were observed at a high rate. In addition, although we had some cases diagnosed with segmental type adenomyoma, there was no case of the diffuse type adenomyoma.

A cholecystectomy rate of 52%-78% has been reported for cholelithiasis materials with adenomyoma (4). There exist some hypotheses that these lesions have a predisposition for the formation of gallstones. It has been reported that biliary stasis in the fundal compartment plays an important role for the lithogenic environment and therefore creates a predisposing condition for gallstones particularly in segmental adenomyomas (4). In our study, the adenomyoma type in the gallstone accompanying cases was the fundal type. Only one of the cases diagnosed with segmental adenomyoma had a gallstone. It has been reported that cholesterolosis may also accompany adenomyomas in 33% of the cases (4). In some studies, it is maintained that cholesterol deposition in the gallbladder wall may cause gallbladder dysfunction (12). This may eventually lead to motility dysfunction and intraluminal pressure increase which play a role in adenomyoma pathogenesis. In the present study, the gallstone and cholesterolosis diagnosis rates were parallel with the ones reported in the literature (4).

Cancer is the most frequent consideration while polyps, lipomas, adenomas, and acute and xanthogranulomatous cholecystitis are also included in the differential diagnosis of adenomyomas (5). Adenomyomas, defined as benign hyperplastic lesions, do not have more neoplastic potential than a normal gallbladder (3). However, diagnostic confusion may arise due to their pseudoinvasive appearance that mimics the morphology of adenocarcinoma. It has also been reported that in situ or invasive carcinomas could emerge (13). In some studies, the carcinoma incidence was reported to be higher especially in gallbladders that have segmental type adenomyomas (14). These results were observed particularly in cholelithiasis cases, which is a well-known predisposing factor for carcinoma in the elderly population (9). Inflammation and chronic irritation that occur due to the gallstones that frequently accompany segmental adenomyomas may act as the first step in the metaplasia-dysplasia and carcinoma sequence (2). In our study, there were no cases of gallbladder carcinoma accompanying the adenomyoma.

The protocols required to be followed in gallbladder sampling and microscopic examination are still very important and they remain a subject of discussion due to the inability of determining high grade dysplasia and even invasive carcinomas with macroscopic evaluation (6). In the literature, pyloric and intestinal metaplasia, low and high grade dysplasia and early carcinoma diagnosis rate increases are reported by longitudinal sampling between the cystic duct surgical margin and the fundus (7,15). We detected an increase in our adenomyoma diagnosis rates in the post-protocol period in our study which examines the contribution of sampling to the detection of adenomyoma. In addition, we observed a significant increase in the detection of intestinal, pyloric metaplasia and low grade dysplasia that accompanied adenomyoma in the post-protocol period.

Conducting a longitudinal sampling between the neck region and fundus according to the new protocol without causing an increase in the number of cassettes and labour rather than doing a sampling in one or two cassettes concerning the fundus, body and neck in the pre-protocol period can ensure evaluation of a larger amount of mucosa and detection of higher numbers of pathology. This method could also ensure standardization and minimize differences and discrepancies in sampling techniques that are dependent on individuals and vary from one individual to another. We therefore recommend that the application of this technique be handled attentively as in our clinics.

In conclusion, although there was no statistically significant difference between the two periods in our study, an increase in the diagnosis of adenomyoma has been observed following the protocol compared to the pre-protocol period. In addition to the increase observed in the findings that accompany adenomyoma in the post-protocol period, the increase in segmental type adenomyomas detected in the post-protocol period is also noteworthy. By creating awareness, our study may contribute to minimizing the discrepancies in the diagnosis and interpretation of adenomyoma. We think that the post-protocol technique can ensure standardization among pathologists in the sampling of the gallbladder, which in return can help to increase the detection of various pathologies as we have observed in our study.

## References

[ref-1] Joshi Jonathan K., Kirk Lindsey (2018). Adenomyomatosis. StatPearls.

[ref-2] Pellino Gianluca, Sciaudone Guido, Candilio Giuseppe, Perna Giuseppe, Santoriello Antonio, Canonico Silvestro, Selvaggi Francesco (2013). Stepwise approach and surgery for gallbladder adenomyomatosis: a mini-review. Hepatobiliary Pancreat Dis Int.

[ref-3] Bonatti Matteo, Vezzali Norberto, Lombardo Fabio, Ferro Federica, Zamboni Giulia, Tauber Martina, Bonatti Giampietro (2017). Gallbladder adenomyomatosis: imaging findings, tricks and pitfalls. Insights Imaging.

[ref-4] Nishimura Atsushi, Shirai Yoshio, Hatakeyama Katsuyoshi (2004). Segmental adenomyomatosis of the gallbladder predisposes to cholecystolithiasis. J Hepatobiliary Pancreat Surg.

[ref-5] Golse N., Lewin M., Rode A., Sebagh M., Mabrut J.-Y. (2017). Gallbladder adenomyomatosis: Diagnosis and management. J Visc Surg.

[ref-6] Adsay Volkan, Saka Burcu, Basturk Olca, Roa Juan Carlos (2013). Criteria for pathologic sampling of gallbladder specimens. Am J Clin Pathol.

[ref-7] Argon Asuman, Yağcı Ayşe, Taşlı Funda, Kebat Tulu, Deniz Senem, Erkan Nazif, Kitapçıoğlu Gül, Vardar Enver (2013). A different perspective on macroscopic sampling of cholecystectomy specimens. Korean J Pathol.

[ref-8] Esendağlı Güldal, Akarca F. Göknur, Balcı Serdar, Argon Asuman, Erhan Selma Şengiz, Turhan Nesrin, Zengin Neslihan İnce, Keser Sevinç Hallaç, Çelik Betül, Bulut Tangül, Abdullazade Samir, Erden Esra, Savaş Berna, Bostan Temmuz, Sağol Özgül, Ağalar Anıl Aysal, Kepil Nuray, Karslıoğlu Yıldırım, Günal Armağan, Markoç Fatma, Saka Burcu, Özgün Gonca, Özdamar Şükrü Oğuz, Bahadır Burak, Kaymaz Esin, Işık Emre, Ayhan Semin, Tunçel Deniz, Yılmaz Banu Özgüven, Çelik Sevinç, Karabacak Tuba, Seven İpek Erbarut, Çelikel Çiğdem Ataizi, Gücin Zuhal, Ekinci Özgür, Akyol Gülen (2018). A Retrospective Evaluation of the Epithelial Changes/Lesions and Neoplasms of the Gallbladder in Turkey and a Review of the Existing Sampling Methods: A Multicentre Study. Turk Patoloji Derg.

[ref-9] Pang Liwei, Zhang Yan, Wang Yuwen, Kong Jing (2018). Pathogenesis of gallbladder adenomyomatosis and its relationship with early-stage gallbladder carcinoma: an overview. Braz J Med Biol Res.

[ref-10] Frierson HF, Stenberg SS (1997). Gallbladder and extrahepatic biliary system. Histology for pathologists.

[ref-11] Runner Gabriel J., Corwin Michael T., Siewert Bettina, Eisenberg Ronald L. (2014). Gallbladder wall thickening. AJR Am J Roentgenol.

[ref-12] Dairi Saif, Demeusy Andrew, Sill Anne M., Patel Shirali T., Kowdley Gopal C., Cunningham Steven C. (2016). Implications of gallbladder cholesterolosis and cholesterol polyps?. J Surg Res.

[ref-13] Adsay V, Mills SE (2010). Gallbladder, extrahepatic biliary tree and ampulla. Stenberg's Diagnostic Surgical Pathology.

[ref-14] Nabatame N., Shirai Y., Nishimura A., Yokoyama N., Wakai T., Hatakeyama K. (2004). High risk of gallbladder carcinoma in elderly patients with segmental adenomyomatosis of the gallbladder. J Exp Clin Cancer Res.

[ref-15] Bolat F, Kayaselçuk F, Nursal TZ, Bal N, Tuncer İ (2007). Kolesistektomilerde örnek sayısının artırılması ile histopatolojik bulguların korelasyonu. Turk Patoloji Derg.

